# Collagenated Synthetic Bone Substitute Material for Sinus Floor Elevation at Sites with a Perforated Schneiderian Membrane

**DOI:** 10.3390/jcm9113764

**Published:** 2020-11-22

**Authors:** Sangyup Kim, Jong-Hyuk Chung, Seung-Yun Shin, Seung-Il Shin, Ji-Youn Hong, Hyun-Chang Lim

**Affiliations:** 1 Department of Periodontology, Graduate School, Kyung Hee University, Seoul 02447, Korea; spata84@hanmail.net; 2 Department of Periodontology, Periodontal-Implant Clinical Research Institute, School of Dentistry, Kyung Hee University, Seoul 02447, Korea; chungjh@khu.ac.kr (J.-H.C.); ssyislet@khu.ac.kr (S.-Y.S.); periohjy@khu.ac.kr (J.-Y.H.)

**Keywords:** collagenated bone substitute material, sinus floor elevation, Schneiderian membrane perforation

## Abstract

Schneiderian membrane perforation (SMP) is the most common complication during sinus floor elevation (SFE). Conventional methods to repair SMP, such as using a collagen barrier, may be clinically demanding. The aim of the present study was to compare the effects of collagenated bone substitute materials with and without a collagen barrier to repair SMP during SFE in terms of new bone formation and dimensional stability. In 12 rabbits, intentional SMP was made during bilateral SFE. The rabbits were randomly assigned under two groups: the control group, in which the sinus was repaired with a collagen barrier, and the test group, in which the sinus was repaired without a collagen barrier. Collagenated bone substitute material was grafted in both groups. Healing periods of 2 weeks and 4 weeks were provided in both groups. There were no adverse clinical events. Histology revealed that the Schneiderian membrane had atrophied with loss of cilia and serous glands in both groups at 4 weeks. Histomorphometry revealed that the newly formed bone (test: 0.42 ± 0.17 mm^2^, control: 0.36 ± 0.18 mm^2^ at 2 weeks; test: 1.21 ± 0.36 mm^2^, control: 1.23 ± 0.55 mm^2^ at 4 weeks) or total augmented area did not significantly differ between the two groups at either time points (*p* > 0.05). In conclusion, collagenated bone substitute material without a collagen barrier demonstrated similar new bone formation and dimensional stability as that with a collagen barrier in repairing SMP.

## 1. Introduction

The posterior maxilla remains a clinically demanding area for implant placement, mainly owing to maxillary sinus pneumatization. Since Boyne introduced sinus floor elevation (SFE) in the 1960s, extensive research has been conducted on this therapeutic modality [1], leading to significant advances in techniques and materials. Several systematic reviews revealed the predictability of SFE and successful long-term outcomes with a high implant survival rate of over 88.64% for 5–10 years [2].

A common surgical complication during SFE is Schneiderian membrane perforation (SMP), with a reported incidence of 0–31.5% [2]. Risk factors for SMP are a history of sinus surgery; sinus pathology; and various anatomical factors, including the Schneiderian membrane thickness, residual bone height, septum, irregular sinus floor, and narrow angle between sinus bone walls [3,4]. The sequelae of SMP include displacement, scattering, and loss of graft materials; postoperative infection (acute and chronic sinusitis); and implant failure [5,6,7,8]. A retrospective study also demonstrated the inverse relationship between the size of SMP and the implant survival rate [9].

Several techniques were introduced to repair SMP, such as using fibrin glue, sutures, collagen barriers, lamellar bone sheathes, pedicled buccal fat pad flaps, and bone block grafts [4,5,9,10]. Among them, collagen barriers of various designs are mostly used for separating the augmented space from the rest of the sinus cavity [11,12], but precisely positioning a collagen barrier on the perforated site requires effort. Occasionally, this is technically demanding and time-consuming.

The possibility of excluding this repairing step for SMP has recently been suggested [13]. In that study, the surgeon only packed the bone substitute material toward the sinus floor side to confine it without placing a collagen barrier. During a mean follow-up period of 11.5 months, no implant failed. Despite the empirical nature of that study, it indicated that when the grafted bone substitute material was stable, augmentation could serve as a proper bed for bone formation, osseointegration, and load-bearing of implants.

Considering the above, collagenated bone substitute material can easily provide graft stability in cases of SMP. Collagenated bone substitute material absorbs blood and can be readily stabilized in a cluster shape in various local conditions.

This study was aimed at comparing the effects of collagenated synthetic bone substitute material with and without a collagen barrier to repair SMP during SFE in terms of new bone formation, dimensional stability, and healing of the perforated Schneiderian membrane.

## 2. Materials and Methods

The protocol of the present preclinical study was approved by the institutional research committee (KHMC-IACUC 19-015). The ARRIVE guidelines for animal research were followed [14]. Animal surgeries were performed in the period from 23 August 2019 to 4 October 2019.

### 2.1. Animals

Twelve New Zealand white male rabbits, weighing 2.5–3.0 kg, were chosen in the present study. The rabbits were housed in individual cages in standard laboratory conditions. They were fed a soft pellet diet and provided with ad libitum access to water. Preoperatively, a >1 week off-period was given to the rabbits to adjust to the laboratory environment. The medical status of the rabbits was regularly monitored.

### 2.2. Study Design

In bilateral maxillary sinuses, the following groups were established. In the test group, SMP was intentionally performed during SFE and grafted with 0.2 cc collagenated synthetic bone substitute material (OSTEON™ III; Genoss, Suwon, Korea) composed of 94% biphasic calcium phosphate and 6% collagen. In the control group, SMP was intentionally performed during SFE and repaired using a native bilayer collagen barrier (Bio-Gide; Geistlich Pharma, Wolhusen, Switzerland). Both groups comprised six rabbits each, and healing periods of 2 weeks and 4 weeks were provided in both groups.

### 2.3. Sample Size Calculation

Sample size was not calculated because of the pilot nature of the present study.

### 2.4. Surgery

All surgeries were performed under general anesthesia induced using an intramuscular injection of a mixture of xylazine hydrochloride (Rompun; Bayer Korea, Seoul, Korea) and Zoletil 50 (Virbac SA; Virbac Laboratories 06516, Carros, France). The surgical site was shaved and disinfected with an iodine solution. Local anesthesia was performed with 2% lidocaine hydrochloride with 1:100,000 epinephrine (Lidocaine HCl; Huons, Seoul, Korea).

A midsagittal incision was made to expose the antral bone. A specially designed sinus step drill kit (SIS Sinus Crestal and Lateral Approach Kit; Shinhung, Seoul, Korea) was used to make an access hole to the sinus cavity. The bony access hole was 4 mm in width and 10 mm in length. The Schneiderian membrane was carefully elevated with a sinus curette (DASK kit; Dentium, Seoul, Korea). SMP did not occur during SFE. Subsequently, the Schneiderian membrane was linearly perforated with a blade in the sagittal direction. The length of the perforation was between 5 mm and 6 mm (approximately half the length of the bony window).

**Figure 1:**
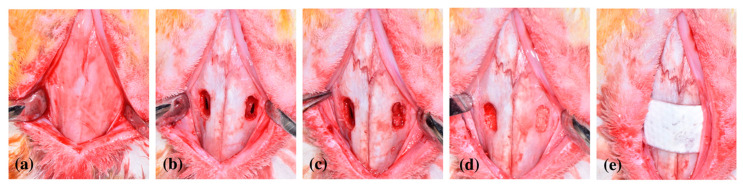
Clinical photographs of the surgery (**a**) after flap elevation, (**b**) during preparation of the bony access windows and perforation of the Schneiderian membrane, (**c**) during placement of a collagen barrier in one sinus, (**d**) during insertion of collagenated synthetic bone substitute material, and (**e**) during closure of the bony window with a cross-linked collagen membrane.

Following intentional SMP, six rabbits were assigned to the test group using a coin-flip and the remaining six rabbits were assigned to the control group. In the test group, 0.2 cc of collagenated synthetic bone substitute material (OSTEON™ III; Genoss, Suwon, Korea) was gently inserted. In the control group, a collagen barrier (Bio-Gide; Geistlich Pharma, Wolhusen, Switzerland) was trimmed to a size of 6 × 12 mm and placed carefully on the perforated site. Subsequently, the same amount of bone substitute material was grafted into the sinus with a repaired SMP. Bilateral bony access holes were covered with a cross-linked collagen membrane (Collagen membrane P, Genoss), and flaps were closed with a 4-0 nylon suture (Blue Nylon; AILEE Comp. Ltd., Busan, Korea; Figure 1).

Postoperatively, the antibiotic gentamycin 0.3 mL (Komi Gentamicin; Komipharm International, Siheung, Korea) and analgesic ketoprofen 0.3 mL (Ketopro, Uni Biotech, Anyang, Korea) were intramuscularly administered for 3 days. The rabbits were carefully monitored in the healing period and sacrificed at 2 (*n* = 6) or 4 (*n* = 6) weeks.

### 2.5. Histologic Processing

Block sections, including the sinuses and adjacent tissues, were harvested and fixed in 10% neutral buffered formalin for 10 days. Fixed specimens were decalcified, trimmed, and embedded in paraffin. The block was sectioned serially at 5 μm in the coronal plane. The most central section of the sinus, including the access hole, was subjected to Masson’s trichrome staining. Stained slides were digitally scanned for histologic and histomorphometric analyses.

### 2.6. Histomorphometric Analysis

Digitally scanned slides were observed using computer software CaseViewer version 2.3 (3DHISTECH, Budapest, Hungary). A blinded investigator (S.K.) performed the histomorphometric analysis with the image analysis software Adobe Photoshop CS6 extended (Adobe Systems, San Jose, CA, USA).

The following parameters were measured: (i) total augmentation (TA): area of total augmentation surrounded by the medial and lateral bony walls, Schneiderian membrane, and surgical access window; (ii) newly formed bone (NB): area of newly formed bone within TA (primary outcome); (iii) residual bone substitute material (RM): area of residual bone substitute material within TA; (iv) %NB: percentage of NB within TA; and (v) %RM: percentage of RM within TA.

Three rectangular regions of interest (ROIs; size: 0.8 mm × 0.8 mm) were set within the augmented area: ROI_W, close to the surgical access window; ROI_C, center of augmentation; and ROI_M, close to the Schneiderian membrane. In ROIs, NB and RM were measured.

### 2.7. Statistics

Statistical analyses were performed using SPSS software version 21.0 (SPSS, Chicago, IL, USA). Data are expressed as mean ± standard deviation and median with interquartile range. Normality of data distribution was tested with the Shapiro–Wilk test. The paired *t*-test or Wilcoxon signed-ranked test was used to compare the test and control groups at each healing time point. The independent *t*-test or Mann–Whitney U test was used to compare parameters between 2 weeks and 4 weeks. A *p*-value < 0.05 was considered to be statistically significant.

## 3. Results

### 3.1. Number of Animals Analyzed

A total of 12 specimens, including six specimens each from the test and control groups, were included for histologic and histomorphometric analyses at 2 weeks and 4 weeks.

### 3.2. Clinical Observations

No adverse event, such as pus discharge, graft popping-up, or swelling, was observed. All rabbits remained healthy in the follow-up period.

### 3.3. Histologic Observation

#### 3.3.1. At 2 Weeks

**Figure 2:**
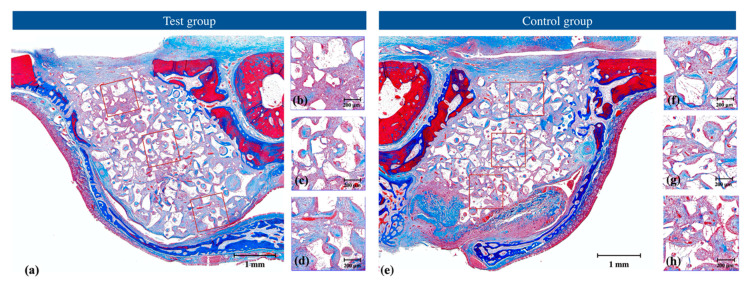
Representative histological views with Masson’s trichrome staining at 2 weeks of the (**a**–**d**) test and (**e**–**h**) control groups showing (**a**,**e**) total augmentation, (**b**,**f**) region of interest close to the surgical access window, (**c**,**g**) region of interest at the center of augmentation, and (**d**,**h**) region of interest close to the Schneiderian membrane.

Dome-shaped augmentation was observed in all specimens. Bone substitute particles were well retained within the sinus cavity over the Schneiderian membrane, except for two specimens in the control group and one specimen in the test group. At those sites in the control group, the collagen barrier incompletely sealed SMP. However, the spilled bone substitute material was limited around perforation sites (Figure 2).

A small amount of NB was observed in both groups, mainly in the area adjacent to the bony access hole. In two specimens in the test group, an NB was found near the Schneiderian membrane (Figure 2).

**Figure 3:**
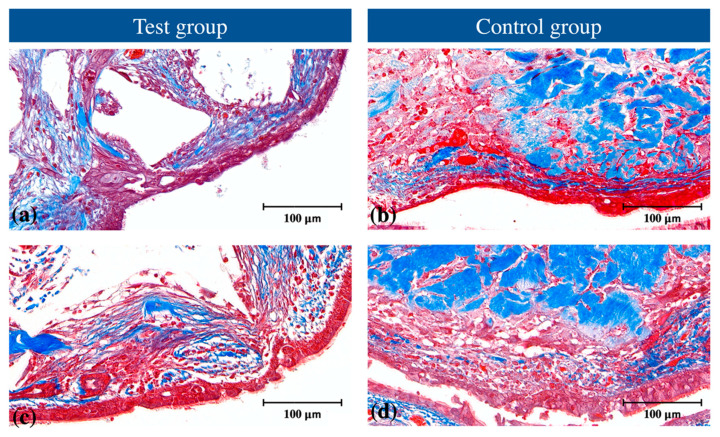
Histological features of the perforated Schneiderian membrane with Masson’s trichrome staining at 2 weeks and 4 weeks in the (**a**) test group at 2 weeks, (**b**) control group at 2 weeks, (**c**) test group at 4 weeks, and (**d**) control group at 4 weeks.

In the SMP area, the Schneiderian membrane was discontinued. Around the perforation, the following were observed: (i) thinner Schneiderian membrane, (ii) erosive epithelial appearance, (iii) loss of cilia, (iv) amorphous goblet cells, and (v) loss of serous glands (Figure 3).

#### 3.3.2. At 4 Weeks

**Figure 4:**
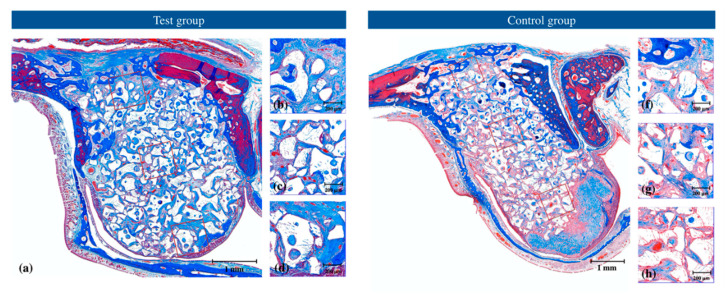
Representative histological views at 4 weeks with Masson’s trichrome staining of the (**a**–**d**) test and (**e**–**h**) control (**a**,**e**) groups showing total augmentation, (**b**,**f**) region of interest close to the surgical access window, (**c**,**g**) region of interest at the center of augmentation, and (**d**,**h**) region of interest close to the Schneiderian membrane.

The appearance of augmentation at 4 weeks was similar to that at 2 weeks. Augmentation had a dome-like shape in both groups at both time points. NB increased from 2 weeks to 4 weeks. In three specimens each in the test and control groups, the access hole area was mostly bridged with NB. A very little bone formation was observed in the center of the augmentation. Near the Schneiderian membrane, varying amounts of NB were observed in most specimens. In both groups, NB near the Schneiderian membrane was greater in one experimental animal compared to others. In the control group, the collagen barrier minimally differed in thickness between 2 weeks and 4 weeks (Figure 4).

Perforated areas appeared to have recovered in continuity at 4 weeks compared to 2 weeks. However, epithelial erosion and loss of cilia/goblet cells persisted. Serous glands were sparse in the area adjacent to the perforated site (Figure 3). The thickness of the Schneiderian membrane adjacent to the perforated area varied without any specific tendency and ranged from 0.034 to 0.162 mm.

### 3.4. Histomorphometric Analysis

Histomorphometric results are presented in Table 1 and Table 2 (absolute values) and Table A1 and Table A2 in Appendix A (percentages). Histologic specimens in the control group demonstrating the spilled bone substitute material were not excluded from the statistical analysis because they reflected a limitation of the treatment modality.

#### 3.4.1. Within the Entire Augmentation

**Figure 5:**
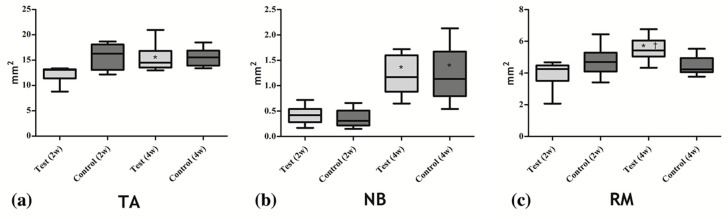
Box plots representing the histomorphometric analysis. (**a**) the area of total augmentation, (**b**) the area of newly formed bone, (**c**) the area of residual bone substitute material, TA: total augmented area, NB: newly formed bone, RM: residual bone substitute material. * Statistically significantly different from 2 weeks. ^†^ Statistically significantly different from the control group.

The area of TA did not significantly differ between the test and control groups at 2 (12.29 ± 1.75 vs. 15.78 ± 2.60 mm^2^) or 4 weeks (15.36 ± 2.88 vs. 15.57 ± 1.83 mm^2^; *p* > 0.05). The area of NB also did not significantly differ between the test and control groups at 2 (0.42 ± 0.17 vs. 0.36 ± 0.18 mm^2^) or 4 weeks (1.21 ± 0.36 vs. 1.23 ± 0.55 mm^2^; *p* > 0.05). In both groups, NB increased from 2 to 4 weeks (*p* < 0.05). The area of RM did not significantly differ between the two groups at 2 weeks (3.94 ± 0.95 vs. 4.75 ± 0.99 mm^2^; *p* > 0.05) but was significantly greater in test group than in the control group at 4 weeks (5.50 ± 0.79 vs. 4.44 ± 0.62 mm^2^; *p* < 0.05; Figure 5).

#### 3.4.2. Region of Interests (ROIs)

In areas adjacent to the bony access window (ROI_W) and at the center of augmentation (ROI_C), the amount of NB was very limited at 2 weeks (mean value < 0.02 mm^2^). In the test group, NB was greater in ROI_M (0.03 ± 0.04 mm^2^) compared to other ROIs. Over time, NB tended to increase in all ROIs, most notably in the ROI_W of both groups (0.07 ± 0.05 in the test group, 0.07 ± 0.04 in the control group at four weeks) (*p* < 0.05 when two and four weeks were compared). RM in ROI_C significantly differed between the test and control groups at 2 weeks (*p* < 0.05) but did not significantly differ in any other comparison (*p* > 0.05).

## 4. Discussion

The present study investigating the effects of collagenated bone substitute material with and without a collagen barrier in the augmented sinus with SMP demonstrated (1) no significant difference in new bone formation or dimensional stability, irrespective of the use of a collagen barrier, and (2) no notable difference in healing of the perforated Schneiderian membrane between the two groups.

Despite the predictability of SFE and technical advancements, several complications are clinically demanding to clinicians, with SMP being the most common. Traditionally, a collagen barrier has been frequently used to repair SMP, but it is time-consuming and technically challenging. The use of collagenated bone substitute material provides cohesive characteristics to the augmented bone owing to the presence of collagen and subsequent absorption of blood [15]. Therefore, such biomaterials may render augmentation more stable even in cases of SMP, which might allow for excluding the collagen barrier.

In the present study, the amount of NB did not significantly differ between the test and control groups at 2 or 4 weeks. The bone-forming rates over the two healing points were similar in both groups. These findings indicated that collagenated synthetic bone substitute material led to similar bone formation capacities in the sinuses with SMP, irrespective of repair using the collagen barrier.

Previously, the effect of a collagen barrier was preclinically tested in the sinuses without SMP [16]. In that study, collagenated bone substitute material derived from the porcine bone was applied. After 2, 4, or 8 weeks of healing, the amount of NB did not significantly differ between the groups with and without the collagen barrier, indicating a negligible effect by the collagen barrier. Another study investigated the role of the collagen barrier in sinus augmentation with SMP [17]. In contrast to a study by Iida et al. (2017), the collagen barrier impaired new bone formation, particularly at 2 weeks of healing. With resorption of the collagen barrier, the amount of NB began to increase from the host bone walls. In addition to SMP, a major difference between the aforementioned studies was the size of the collagen barrier; in the study by Lim et al. (2018), the space created by Schneiderian membrane elevation was almost surrounded by the collagen barrier, resulting in physical separation of the host bone walls from the bone graft material. In the present study, the collagen barrier was confined to the perforated area such that the healing source from the host bone walls was not blocked.

Recently, the effect of collagenated bovine bone mineral without further use of a collagen barrier was tested in a sinus with SMP [18]. In that study, the amount of NB did not significantly differ between the sinuses with and without perforation, supplementing the results of the present study. Collectively, new bone formation in the augmented sinus with SMP could successfully proceed with collagenated bone substitute material. As previously described, exclusion of the repair procedure is advantageous in terms of ease of surgical correction.

One of the major interests in the present study was associated with dimensional stability. In the presence of a Schneiderian membrane perforation, one can assume scattering and displacement of the bone substitute material through the perforation or unfavorable shape of the augmentation owing to uneven Schneiderian membrane elevation. In most histologic specimens in the present study, bone substitute material was well confined. In addition, the total dimension of augmentation did not significantly differ between the test and the control groups. In line with this, similar dimension stability was radiographically and histomorphometrically noted between the sinuses with and without perforation in the study by Paik et al. [18]. The unfavorable shape of augmentation should be noted at 2 weeks in a few specimens in both groups (one in the test group and two in the control group), which presented with bone substitute material slightly spilled via the perforated site. In the control group, the collagen barrier seemed to be displaced, possibly during the insertion of the bone substitute material. However, no noteworthy inflammation was found in those three specimens compared to others.

Interesting findings were demonstrated in a retrospective clinical study [13]. In a case of SMP, the perforated area was blocked using a Prichard elevator, and bone graft particles were compacted toward the sinus floor. After a follow-up period of 11.6 months, no implant failed in the sinuses with SMP. Moreover, the stable height of the augmented bone and no pathologic change in the Schneiderian membrane were observed. That study might imply that stabilization of bone substitute material is essential for the success of the sinus augmentation procedure when SMP occurs. However, with particulate bone substitute material, the graft may be hard to stabilize, particularly by an inexperienced clinician. Moreover, if larger perforation had occurred, the possibility of particulate bone scattering and displacing out of the Schneiderian membrane would be higher. As shown in the present study, collagenated bone substitute material is preferable in terms of graft stabilization over conventional particulate bone material.

The healing of the Schneiderian membrane is of interest. In the present study, the damaged Schneiderian membrane presented with a few serous glands, sparse flattened goblet cells, and atrophied cilia, similar to a previous study [19]. Between 2 weeks and 4 weeks, the membrane regained continuity, but those degenerative features remained. In one study, a perforated Schneiderian membrane was repaired using a collagen barrier and platelet-rich fibrin and histomorphometrically evaluated at 2 weeks and 4 weeks. Compared to an untreated membrane, those materials improved cellular response, angiogenesis, and synthesis of collagen fibrils [20]. However, in the present study, no distinct difference was found between the test and control groups. In another study, observed up to 12 weeks, the perforated Schneiderian membrane healed with dense connective tissue [18]. Taken together, a perforated Schneiderian membrane appears to heal to a clinically acceptable level with augmentation using collagenated bone substitute material.

Followings should be considered in the present study. First, the size of the perforation might have been enlarged during the insertions of the collagen membrane and the graft material. Second, the results of the present study may be only generalized to the small SMP. In case of larger SMP, further studies are required. Third, a clinical study should be supplemented for fully supporting the results of the present study.

## 5. Conclusions

Within the limitations of this study, in the sinuses with a perforated Schneiderian membrane, collagenated bone substitute resulted in similar dimensional stability and new bone formation, irrespective of repair using a collagen barrier. However, clinical evidence should be further supplemented for safe and predictable clinical outcomes.

## Figures and Tables

**Table 1:** Histomorphometric analyses in the test and control groups.

Parameter	Healing Period	Test (*n* = 6 for Each Week)	Control (*n* = 6 for Each Week)	*p*-Value (Test vs. Control)
TA (mm^2^)	2 weeks	12.29 ± 1.75 13.09 (12.47, 13.12)	15.78 ± 2.60 16.27 (13.90, 17.72)	0.082
	4 weeks	15.36 ± 2.88 14.52 (13.83, 15.32)	15.57 ± 1.83 15.55 (14.30, 16.32)	0.87
	*p*-value (2 weeks vs. 4 weeks)	0.026	0.875	
NB (mm^2^)	2 weeks	0.42 ± 0.17 0.42 (0.33, 0.48)	0.36 ± 0.18 0.31 (0.25, 0.43)	0.566
	4 weeks	1.21 ± 0.36 1.17 (0.98, 1.50)	1.23 ± 0.55 1.14 (0.92, 1.45)	0.953
	*p*-value (2 weeks vs. 4 weeks)	0.003	0.004	
RM (mm^2^)	2 weeks	3.94 ± 0.95 4.25 (4.03, 4.39)	4.75 ± 0.99 4.70 (4.40, 4.87)	0.325
	4 weeks	5.50 ± 0.79 5.42 (5.31, 5.71)	4.44 ± 0.62 4.23 (4.16, 4.63)	0.038
	*p*-value (2 weeks vs. 4 weeks)	0.011	0.538	

Data are expressed as mean ± standard deviation and median (quartiles). TA: area of total augmentation surrounded by the medial and lateral bony walls, Schneiderian membrane, and surgical access window; NB: area of newly formed bone within TA; RM: area of residual bone substitute material within TA.

**Table 2:** Amount of newly formed bone and residual bone substitute material in regions of interest (ROI = 0.64 mm^2^).

**ROI_W**	**Healing Period**	**Test** **(*n* = 6 for Each Week)**	**Control** **(*n* = 6 for Each Week)**	***p*-Value** **(Test vs. Control)**
NB (mm^2^)	2 weeks	0.0 ± 0.0 0.0 (0.0, 0.0)	0.01 ± 0.02 0.0 (0.0, 0.0)	0.317
	4 weeks	0.07 ± 0.05 0.08 (0.03, 0.10)	0.07 ± 0.04 0.09 (0.04, 0.10)	1.0
	*p*-value (2 weeks vs. 4 weeks)	0.002	0.009	
RM (mm^2^)	2 weeks	0.21 ± 0.05 0.21 (0.18, 0.25)	0.23 ± 0.05 0.22 (0.20, 0.25)	0.384
	4 weeks	0.20 ± 0.03 0.19 (0.17, 0.23)	0.22 ± 0.07 0.22 (0.20, 0.27)	0.475
	*p*-value (2 weeks vs. 4 weeks)	0.55	0.727	
**ROI_C**	**Healing Periods**	**Test** **(*n* = 6 for Each Week)**	**Control** **(*n* = 6 for Each Week)**	***p*-Value** **(Test vs. Control)**
NB (mm^2^)	2 weeks	0.0 ± 0.0 0.0 (0.0, 0.0)	0.0 ± 0.0 0.0 (0.0, 0.0)	0.317
	4 weeks	0.01 ± 0.01 0.01 (0.0, 0.01)	0.01 ± 0.01 0.01 (0.0, 0.01)	1.0
	*p*-value (2 weeks vs. 4 weeks)	0.31	0.065	
RM (mm^2^)	2 weeks	0.25 ± 0.03 0.23 (0.22, 0.27)	0.29 ± 0.05 0.29 (0.27, 0.30)	0.012
	4 weeks	0.30 ± 0.03 0.31 (0.28, 0.32)	0.29 ± 0.04 0.29 (0.27, 0.31)	0.378
	*p*-value (2 weeks vs. 4 weeks)	0.03	0.742	
**ROI_M**	**Healing Periods**	**Test** **(*n* = 6 for Each Week)**	**Control** **(*n* = 6 for Each Week)**	***p*-Value** **(Test vs. Control)**
NB (mm^2^)	2 weeks	0.03 ± 0.04 0.01 (0.00, 0.04)	0.0 ± 0.0 0.0 (0.0, 0.0)	0.109
	4 weeks	0.04 ± 0.03 0.04 (0.02, 0.05)	0.02 ± 0.04 0.00 (0.00, 0.02)	0.345
	*p*-value (2 weeks vs. 4 weeks)	0.538	0.394	
RM (mm^2^)	2 weeks	0.23 ± 0.07 0.25 (0.20, 0.27)	0.27 ± 0.06 0.25 (0.23, 0.30)	0.219
	4 weeks	0.25 ± 0.05 0.24 (0.21, 0.26)	0.29 ± 0.03 0.29 (0.27, 0.31)	0.206
	*p*-value (2 weeks vs. 4 weeks)	0.532	0.458	

Data are expressed as mean ± standard deviation and median (quartiles). ROI_W: area close to the surgical access window; ROI_C: area at the center of augmentation; ROI_M: area close to the Schneiderian membrane; NB: area of newly formed bone within the ROI; RM: area of residual bone substitute material within the ROI.

## Appendix A

**Table A1:** Percentage of newly formed bone and residual bone substitute material in the total augmented area.

Parameter	Healing Period	Test (N = 6 for Each Week)	Control (N = 6 for Each Week)	*p*-Value (Test vs. Control)
%NB	2 weeks	3.69 ± 2.38 3.21 (2.63, 3.64)	2.18 ± 0.88 2.16 (1.56, 2.43)	0.249
	4 weeks	7.81 ± 2.20 7.14 (6.80, 9.06)	7.71 ± 3.01 7.50 (6.26, 8.09)	0.954
	*p*-value (2 weeks vs. 4 weeks)	0.011	0.002	
%RM	2 weeks	31.62 ± 4.40 32.62 (30.77, 34.63)	30.31 ± 5.63 28.68 (25.91, 33.37)	0.696
	4 weeks	36.08 ± 2.99 36.28 (33.77, 38.47)	28.51 ± 1.44 28.73 (27.92, 29.61)	0.002
	*p*-value (2 weeks vs. 4 weeks)	0.067	0.937	

Data are expressed as mean ± standard deviation and median (quartiles). NB: percentage of newly formed bone within the total augmented area; RM: percentage of residual bone substitute material within the total augmented area.

**Table A2:** Percentage of newly formed bone and residual bone substitute material in the regions of interest (ROIs = 0.64 mm^2^).

**ROI_W**	**Healing Period**	**Test** **(*n*= 6 for Each Week)**	**Control** **(*n* = 6 for Each Week)**	***p*-Value** **(Test vs. Control)**
%NB	2 weeks	0.00 ± 0.00 0.00 (0.00, 0.00)	0.01 ± 0.03 0.00 (0.00, 0.00)	0.317
	4 weeks	0.11 ± 0.07 0.12 (0.05, 0.16)	0.12 ± 0.06 0.13 (0.06, 0.16)	0.915
	*p*-value (2 weeks vs. 4 weeks)	0.002	0.009	
%RM	2 weeks	0.33 ± 0.08 0.32 (0.28, 0.38)	0.36 ± 0.08 0.35 (0.31, 0.39)	0.405
	4 weeks	0.31 ± 0.05 0.30 (0.27, 0.35)	0.34 ± 0.11 0.35 (0.31, 0.42)	0.491
	*p*-value (2 weeks vs. 4 weeks)	0.585	0.716	
**ROI_C**	**Healing Period**	**Test** **(*n* = 6 for Each Week)**	**Control** **(*n* = 6 for Each Week)**	***p*-Value** **(Test vs. Control)**
%NB	2 weeks	0.00 ± 0.00 0.00 (0.00, 0.00)	0.00 ± 0.00 0.00 (0.00, 0.00)	0.317
	4 weeks	0.01 ± 0.02 0.01 (0.01, 0.01)	0.01 ± 0.01 0.01 (0.01, 0.02)	0.783
	*p*-value (2 weeks vs. 4 weeks)	0.132	0.015	
%RM	2 weeks	0.38 ± 0.06 0.36 (0.34, 0.42)	0.45 ± 0.07 0.45 (0.42, 0.47)	0.016
	4 weeks	0.47 ± 0.05 0.48 (0.43, 0.51)	0.45 ± 0.06 0.45 (0.42, 0.49)	0.474
	*p*-value (2 weeks vs. 4 weeks)	0.033	0.838	
**ROI_M**	**Healing Period**	**Test** **(*n* = 6 for Each Week)**	**Control** **(*n* = 6 for Each Week)**	***p*-Value** **(Test vs. Control)**
%NB	2 weeks	0.04 ± 0.06 0.00 (0.00, 0.07)	0.00 ± 0.00 0.00 (0.00, 0.00)	0.180
	4 weeks	0.06 ± 0.04 0.06 (0.03, 0.08)	0.03 ± 0.06 0.0 (0.0, 0.02)	0.345
	*p*-value (2 weeks vs. 4 weeks)	0.394	0.394	
%RM	2 weeks	0.35 ± 0.10 0.39 (0.31, 0.43)	0.42 ± 0.09 0.40 (0.37, 0.46)	0.22
	4 weeks	0.39 ± 0.07 0.37 (0.33, 0.41)	0.45 ± 0.05 0.45 (0.42, 0.48)	0.195
	*p*-value (2 weeks vs. 4 weeks)	0.526	0.446	

Data are expressed as mean ± standard deviation and median (quartiles). ROI_W: area close to the surgical access window; ROI_C: area of the center of augmentation; ROI_M: area close to the Schneiderian membrane; %NB: percentage of newly formed bone within the ROI; RM: percentage of residual bone substitute material within the ROI.
